# Immediate Implant Therapy with Full-Digital Workflow to Replace a Central Incisor

**DOI:** 10.3390/dj13020073

**Published:** 2025-02-08

**Authors:** Francisco Garcia-Torres, Carlos A. Jurado, Silvia Rojas-Rueda, Clint Conner, Ali Abulkasim Mohamed, Francisco X. Azpiazu-Flores

**Affiliations:** 1 Department of Prosthodontics and Implantology, School of Dentistry, University of La Salle, Leon 37150, Mexico; 2 Division of Operative Dentistry, Department of General Dentistry, Health Science Center, College of Dentistry, The University of Tennessee, Memphis, TN 38103, USA; 3 School of Dental Medicine, Ponce Health Sciences University, Ponce 00716, Puerto Rico; 4 Division of Dental Biomaterials, School of Dentistry, The University of Alabama at Birmingham, Birmingham, AL 35233, USA; 5 Division of Restorative and Prosthetic Dentistry, College of Dentistry, The Ohio State University, Columbus, OH 43210, USA

**Keywords:** immediate implant, digital workflow, CAD/CAM, scanners

## Abstract

**Background:** Replacing a maxillary central incisor and immediately placing an implant represents a clinical challenge. **Methods:** This case report demonstrates a full digital workflow to achieve a predictable implant placement and esthetically pleasing restoration for a 35-year-old male patient who suffered a horizontal root fracture after a sports accident. The patient’s treatment included digital implant planning, minimally traumatic tooth extraction, computer-guided implant placement, soft tissue augmentation, and a provisional restoration to contour the augmented gingival tissues. The process began with cone beam computed tomography (CBCT) evaluation and patient consultation on treatment options, with the patient opting for implant therapy. Using a 3D-printed surgical guide, the implant was placed precisely, and a soft tissue graft was used to enhance the gingival architecture and volume. A provisional restoration was designed to support the emergence profile and condition the peri implant soft tissues. A final digital impression was made, and a screw-retained all-ceramic crown was fabricated uneventfully after healing. **Results:** This digital approach allowed accurate planning and allowed the efficient execution of a technique-sensitive procedure such as immediate implant placement, thus providing an esthetic and functional solution while minimizing treatment time. **Conclusions:** The case highlights that immediate implant therapy in the esthetic zone requires meticulous planning and execution, and that incorporating advanced digital tools and techniques is required to achieve favorable clinical outcomes.

## 1. Introduction

Dental trauma can affect all age groups and genders, and its incidence has been recognized as an important problem worldwide with studies suggesting that around 900 million people have experienced damage to their permanent dentition [1]. Epidemiological studies have also indicated an annual incidence of dental trauma of about 4.5%, and approximately one-third of children, and one-fifth of adolescents and adults have experienced a traumatic dental injury [2]. Maxillary central incisors have been identified as the most affected teeth in cases of trauma because they are located anteriorly in the mouth. Therefore, some studies have described that central incisors account for more than 50% of all dental injuries [3,4].

Treating the maxillary anterior teeth represents a challenging clinical scenario for the clinician because small imperfections are easily noticed by the patient. Several factors need to be evaluated, such as the smile line, buccal corridors, the amount of gingival exposure while smiling, the presence of spaces between teeth, teeth proportions, and symmetry, among others [5,6,7]. If the patient presents with hopeless teeth, the clinician may need to evaluate more complex dental procedures, such as implant therapy combined with fixed and removable dental prostheses [8,9].

Implant therapy has been demonstrated to be a great treatment for replacing hopeless teeth in the esthetic zone. Implants can be placed immediately after tooth extraction, at an early placement protocol between one week and two months, or following a delayed placement protocol after two months. The occlusal loading can also be similarly classified by the timing [10]. However, some factors have been described as critical for the timing of implant placement, such as the patient’s health condition, patient habits, management of the extraction site, bone quality, anticipation of socket healing, implant positioning and stability, and control of occlusal forces [11,12]. Immediate implant therapy has been reported to have highly positive outcomes, with a success rate of 96% after five years in posterior sites and 95.5% after five years in anterior sites [13,14].

Digital dental workflows allow clinicians to fabricate clinically acceptable restorations faster and consistently [15]. Moreover, some studies suggest that most patients’ prefer digital workflows over traditional methods [16]. Currently, cone beam computed tomography (CBCT) and scanners are commonly used by clinicians to three-dimensionally plan the implant position and create a precise surgical guide for implant placement. Studies have shown that implant guides fabricated with a digital workflow offer higher accuracy than implants placed with traditional thermoplastic guides [17].

Restorative care involving a single immediate implant in the esthetic zone represent a clinical challenge because several factors need to be considered to achieve predictable results. The present case report presents all steps for the proper planning and execution of replacing a hopeless maxillary right central incisor with an immediate implant and immediate provisionalization to contour the gingival architecture for the final restoration. Moreover, the entire treatment was provided using a digital workflow, allowing clinicians and the patient to obtain the desired outcome.

## 2. Materials and Methods

**Figure 1:**
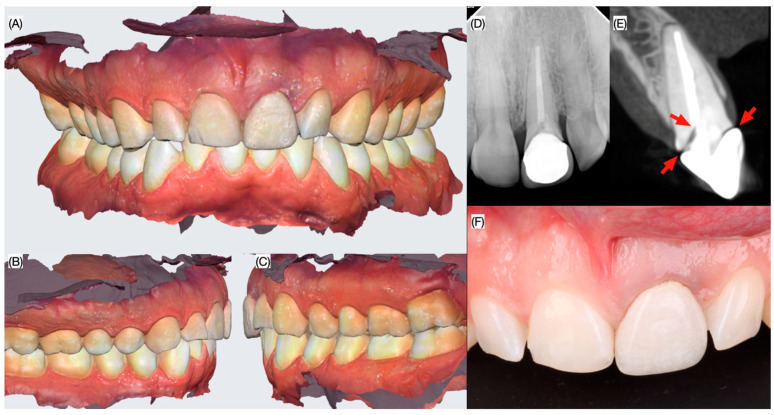
Initial situation. Intra-oral scan (**A**) frontal, (**B**) right and (**C**) left side view, (**D**) peri-apical radiograph, (**E**) interproximal view with CBCT scan showing the fractured lines and (**F**) frontal view.

A 34-year-old male patient presented with the chief complaint “I have mobility in my front crown and I want to fix it”. Patient reported to have suffered trauma from a soccer ball during a game a few days ago, but referred no pain. The medical history revealed no allergies and the patient expressed to be healthy. As a result, the patient was classified as an ASA type I. The clinical evaluation revealed that patient has an all-ceramic crown on tooth #21 with Miller’s mobility class II, and incisal wear on tooth #11. Additionally, the zenith positions of the anterior teeth #13, 12, 11, 21, 22 and 23 was asymmetric. A periodontal evaluation revealed healthy periodontal status and thick biotype and patient claimed to have good oral hygiene and receive dental prophylaxis twice a year. Cone beam computed tomography (CBCT) showed the maxillary right central incisor having a metal post and a horizontal root fracture immediately apical to the margins of the crown thus compromising the restorability of the tooth (Figure 1).

**Figure 2:**
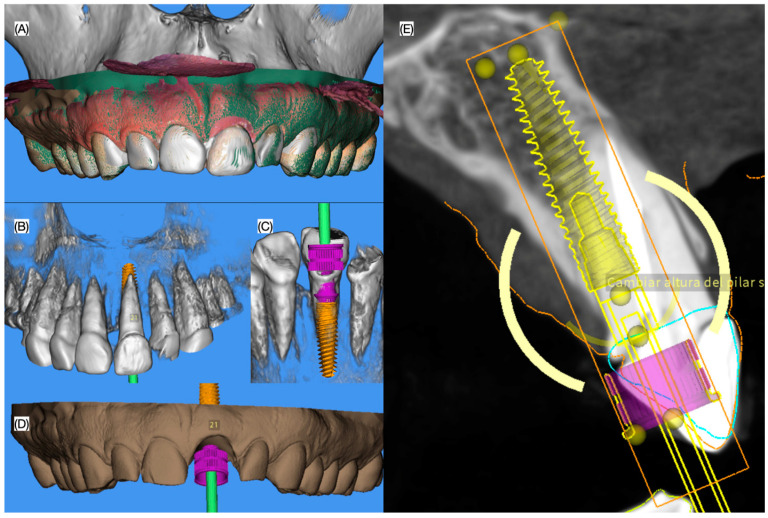
Three-dimensional planning of the implant surgery. (**A**) intra-oral scan overlaying the CBCT image, (**B**) frontal and (**C**) lingual view of the implant planning on the tooth area, (**D**) frontal view of the implant position without the existing tooth and (**E**) interproximal view of the implant planning.

**Figure 3:**
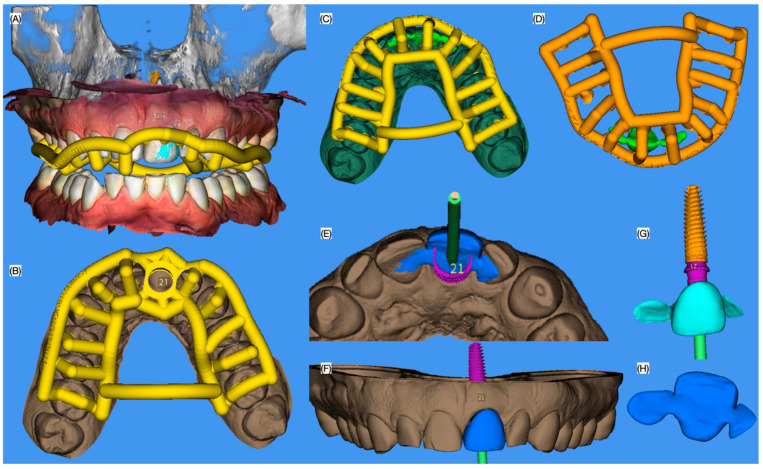
Three-dimensional planning of the surgical and restoration guides, and interim restoration. Digital design of the surgical guide (**A**) frontal and incisal (**B**) view, digital design of the seating guide for the provisional restoration (**C**) with and (**D**) without maxillary arch, design of the provisional restoration (**E**) incisal, frontal (**F**) with and (**G**) without maxillary arch and (**H**) lingual view.

The patient was offered several treatment options including implant therapy, tooth supported fixed dental restoration, and removable partial denture. The patient was also offered crown lengthening in order to improve the gingival architecture, veneers from right to left canine or simply direct resin composite for right central incisor to address the incisal wear. The patient declined the crown lengthening and the veneers but approved the direct resin composite restoration for the right central incisor combined with an implant to replace the left central incisor. Intra-oral scanning (Medit i600, Seoul, Republic of Korea) was performed, and was integrated to the previously taken CBCT to digitally design (MSOFT, MIS Dental Implants, Misgav, Israel) an implant surgical guide and a positioning guide for a provisional restoration. Subsequently, the guide and positioning device were fabricated with a 3-D printer (Phrozen Sonic Mighty 4K Resin 3D Printer, Phrozen Technology, Hsinchu City, Taiwan) using a biocompatible resin (Keyguide Keyprint, Keystone Industries, Gibbstown, NJ, USA) (Figure 2 and Figure 3).

Additionally, an interim restoration with supporting wings was 3D printed (NextDent C&B Micro Filled Hybrid, NextDent, Soesterberg, The Netherlands), and a 3D-printed model of the maxilla was also fabricated using the same 3D printer.

**Figure 4:**
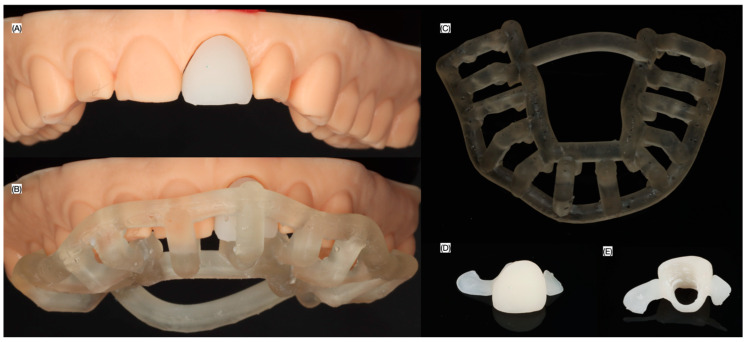
Seating guide and interim restoration. (**A**) Interim restoration on 3D-printed model (**B**) seating guide on 3D-printed model, (**C**) 3D printed guide occlusal view, and interim restoration (**D**) frontal and (**E**) lingual views.

A minimally traumatic tooth extraction was performed with forceps to avoid fracturing of the buccal and lingual cortical plates. After the fresh extraction socket was cleaned and irrigated, an implant (MIS C1 3.75 × 16 mm, MIS Dental Implants, Misgav, Israel) was inserted using the 3D-printed the surgical guide (Figure 4).

**Figure 5:**
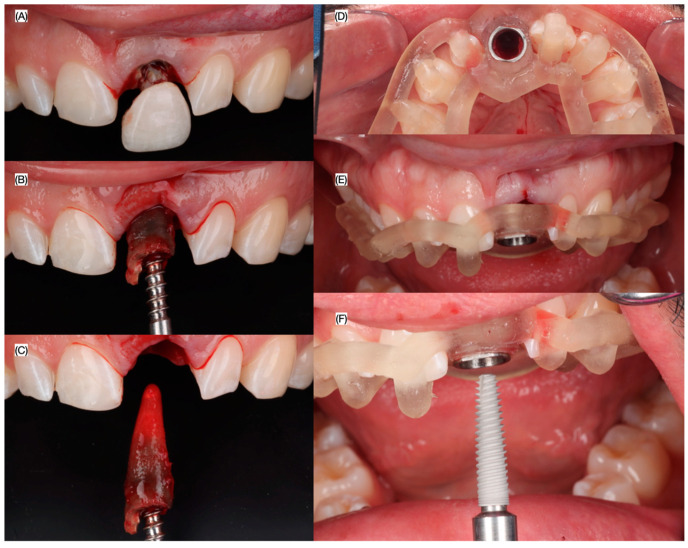
Minimally atraumatic tooth extraction and implant placement. (**A**) Fractured crown removal, (**B**) initial and (**C**) final root removal, (**D**) occlusal view of 3D-printed guide and (**E**) frontal view and (**F**) frontal view of guide used for implant placement.

Primary stability was achieved and the winged-supported 3D-printed restoration. was connected to the implant using flowable composite and a polyetheretherketone (PEEK) abutment (Direct Temporary Abutment, MIS Implants, Misgav, Israel). The restoration was left without any occlusal contacts to prevent occlusal overloading (Figure 5). Subsequently, a subepithelial connective tissue graft harvested from the palate was placed in the facial surface of the tissue to improve the gingival contours. Moreover, cancellous granular xenograft material (Geistlich, Bio-Oss, Wolhusen, Switzerland) was placed to close the jumping gap between implant and buccal plate.

**Figure 6:**
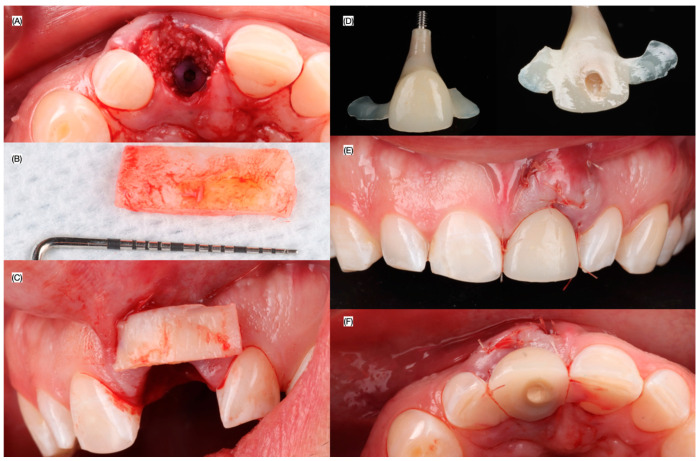
Grafting and interim restoration. (**A**) Incisal view after bone grafting, (**B**) size of the connective tissue, (**C**) intraoral evaluation of the connective tissue to be placed, (**D**) facial and lingual view of screw retained interim restoration, provisional restoration inserted (**E**) frontal view, and (**F**) incisal view.

**Figure 7:**
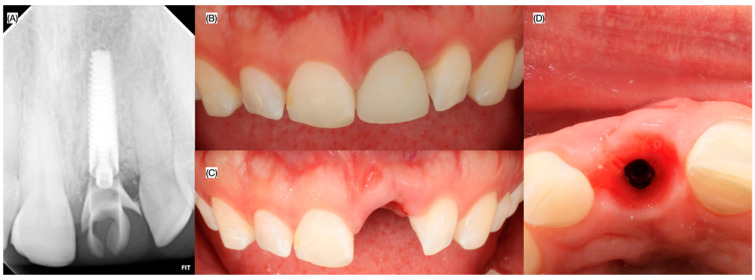
Follow-up evaluation. (**A**) Periapical radiograph, frontal view at the fourth month follow-up (**B**) with and (**C**) without provisional restoration, and (**D**) incisal view without provisional restoration.

**Figure 8:**
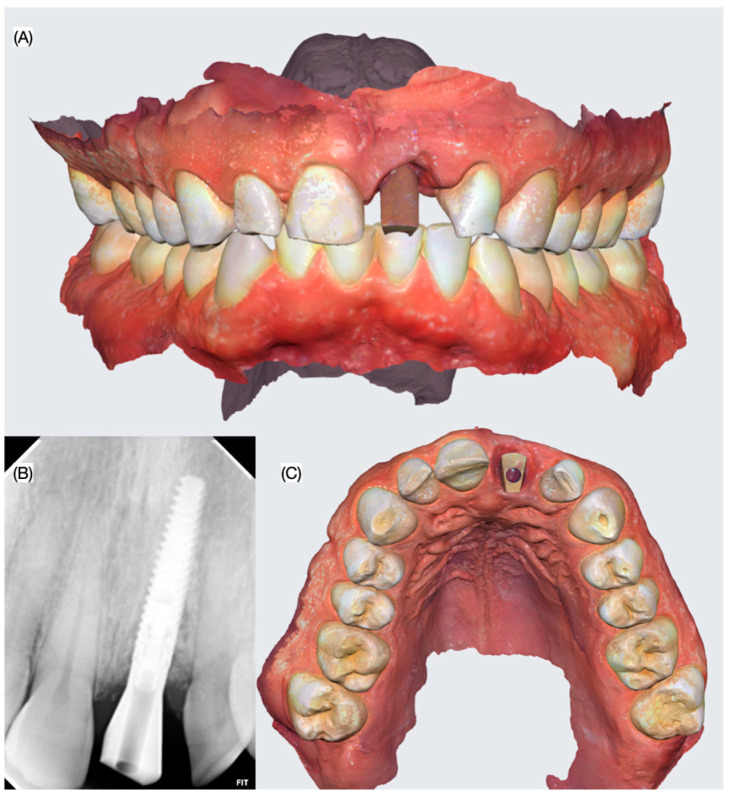
Final digital impression. (**A**) Frontal view, (**B**) periapical radiograph and (**C**) incisal view of the scan body used for digital impression.

Patient received follow-up evaluation 24 h, 1, 2 and 4 weeks, 2 and 3 months after the surgery. A new provisional restoration was placed at the 3 months to finalize contouring the gingival tissues, and a final digital impression (Medit i600, Seoul, Republic of Korea) was made at the fourth month, in conjunction with intra-oral photos (Nikon d7500, Nikon, Tokyo, Japan) without and with the cross polarized lens to eliminates glare and to assess the translucency for shade matching of the ceramic restoration (Figure 6, Figure 7 and Figure 8).

**Figure 9:**
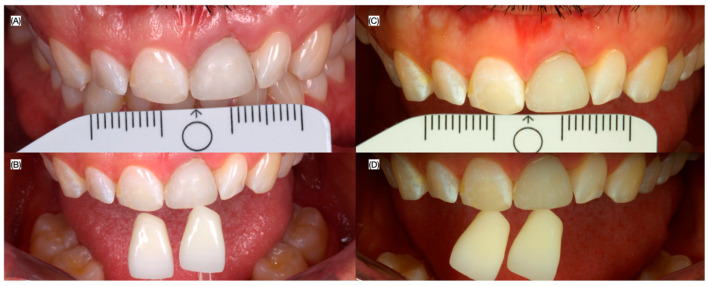
Intra-oral photographs used for shade-matching. Photographs with (**A**) white calibration card and with (**B**) shade tabs, and cross polarized photos with (**C**) white calibration card and with (**D**) shade tabs.

A layered-zirconia (Prettau 5 Anterior Disperse, Zirkonzahn GmbH, Gais, Italy) screw-retained crown was fabricated and cemented (Panavia V5 LC, Kuraray Noritake, Tokyo, Japan) to a prefabricated titanium based (Ti-Base, Mis Implants, MIS Implants, Misgav, Israel). The implant restoration was placed intraorally, and immediately, a nano resin composite (Clearfill AP-X-ES-2, Kuraray, Tokyo, Japan) was applied at the incisal edge of the maxillary right central incisor in order to match the length of the implant restoration. The patient was pleased with the contours, shade and shape of the implant supported crown, and the resin composite restorations. (Figure 9).

**Figure 10:**
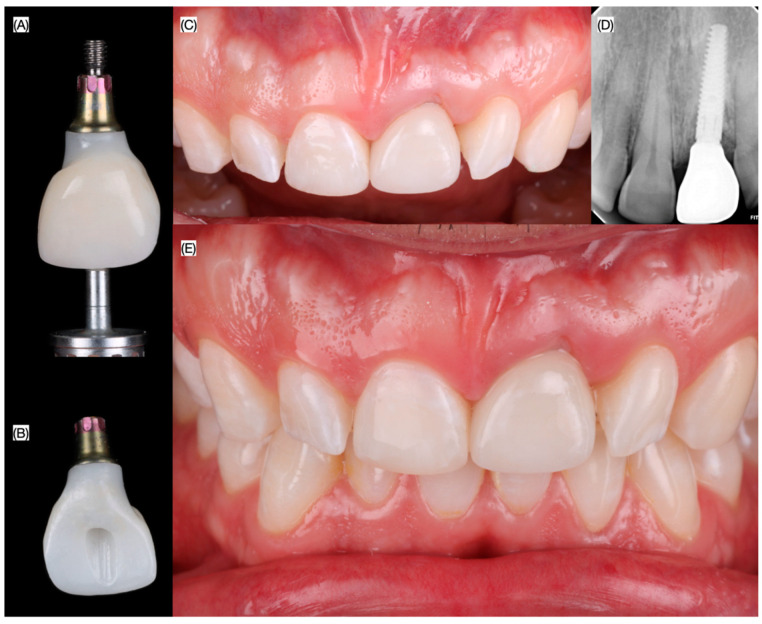
Final restorations. Screw retained implant provisional (**A**) frontal and (**B**) lingual view prior placement, (**C**) intra-oral frontal view, (**D**) radiograph, and (**E**) frontal intraoral photograph occlusion.

At the time of delivery, the patient received an occlusal guard to wear at night to protect the restorations (Figure 10).

**Figure 11:**
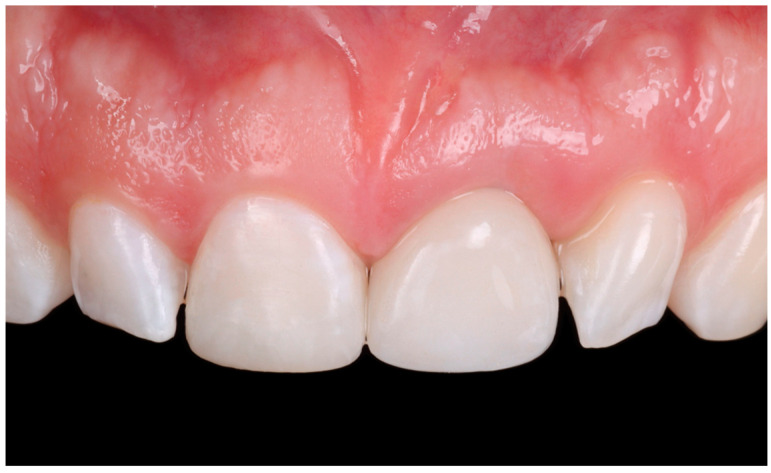
Follow-up evaluation of the restorations.

At the three-year follow-up evaluation, the implant and resin composite restorations were stable, and the patient was pleased with the esthetic outcomes (Figure 11).

## 3. Results

A thoughtful clinical assessment is needed whenever an immediate implant is selected to replace a hopeless tooth in the esthetic zone. The first step includes a CBCT evaluation to assess the amount of bone available buccally and lingually. This allows the clinician to select the width and length of the implant that fits better the clinical scenario. Once digital planning is completed, a printed surgical guide is created to place the implant in the digitally planned position. Soft tissue grafting at the time of implant placement prevents gingival recessions and helps to maintain the gingival volume around the implant. This is accompanied with a provisional restoration that helps develop the gingival architecture to achieve an emergence profile that matches the adjacent natural dentition.

**Figure 12:**
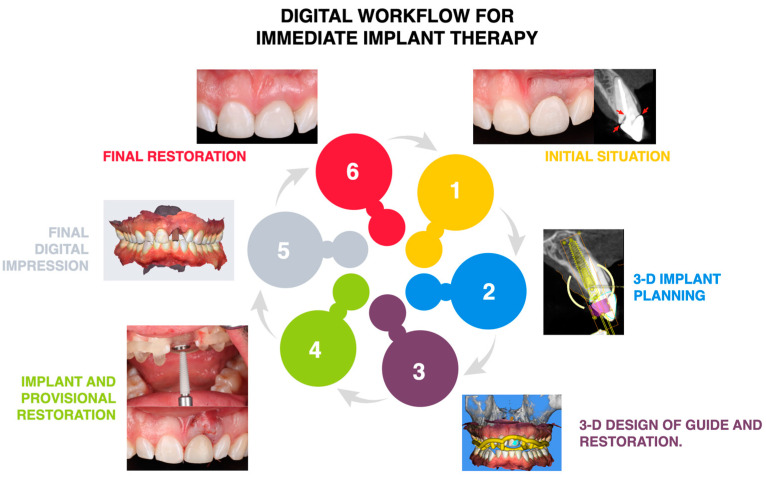
Summary of the study workflow.

In the present article, the digital workflow implemented permitted the design of the restoration with the desired width and length to match the adjacent teeth, which is fundamental when restoring a single central incisor. The patient was satisfied with the final result, and at the three-year follow-up appointment, the restoration and tissue continued to meet the patient’s esthetic and functional demands. A flowchart describing the step for the digital workflow implemented can be seen in Figure 12.

## 4. Discussion

The implant therapy presented in this article allowed successfully replacing a fractured, hopeless maxillary right central incisor. The three-dimensional evaluation enabled the assessment of the bone available to place the implant. A minimally traumatic tooth extraction preserved the buccal and lingual plates, providing a favorable prognosis for the implant. Moreover, soft tissue grafting procedures, in conjunction with the implant placement allowed preventing gingival recessions and maintaining ideal gingival contours. The recommended clinical scenarios for achieving ideal clinical outcomes are described in Table 1 [18,19,20].

The success of immediate implants has been well-documented in the literature. A systematic review evaluating the survival of single immediate implants and reasons for loss initially identified 6042 studies, screened 364, and ultimately reviewed 28 studies. These studies provided information on implants placed with at least a 12-month follow-up, and the results indicated a survival rate ranging from 83.7% to 100%. The reasons for failure included infection, mobility after immediate loading, and iatrogenic complications [21]. Moreover, a recent in vivo study evaluated the clinical success of immediate loading implants in the esthetic zone. This study placed 20 implants in 20 patients and evaluated them one week after surgery and at one-, three-, and six-month follow-ups to assess mobility and soft tissue conditions. All the implants were successful at the one- and three-month follow-ups, but three implants exhibited mobility at the sixth-month follow-up. Nineteen patients were either very or fairly satisfied, and the authors concluded that immediate implant placement in the esthetic zone achieved a high success rate and good patient acceptance [22]. Due to the positive data presented in the literature, immediate implant therapy was provided in the present case study.

Soft tissue grafting procedures have been shown to be a positive treatment in conjunction with immediate implant therapy. A recent systematic review and meta-analysis evaluated the use of tissue grafts associated with immediate implant placement to achieve better peri-implant stability and efficacy. The review initially identified 260 articles, screened 16, and ultimately included 9 randomized clinical trials. It concluded that using bone and soft tissue grafting techniques associated with immediate implant placement prevents significant tissue reduction and provides greater bone stability and higher esthetic levels [23]. Moreover, recent results from a 5-year randomized controlled trial evaluating single immediate implant placement in the maxillary esthetic zone with and without connective tissue grafting assessed 60 patients with a single failing tooth. Immediate implants were placed with either a connective tissue graft from the maxillary tuberosity or without grafting. The implants were assessed at 1, 12, and 60 months after final crown placement, and the results showed a 96.7% survival rate for both groups and fewer tissue changes (−0.4 to 0.5 mm) in implants with grafting procedures than in implants without grafting (−1.1 to −0.1 mm). The authors concluded that grafting connective tissue with immediate implant placement results in favorable peri-implant tissue with fewer changes [24]. Due to the positive outcomes, the present case report also included the combination of soft tissue grafting with immediate implant placement.

The full digital workflow shown in this clinical report included a final impression in the anterior zone with an intra-oral scanner. A recent systematic review evaluated the accuracy of digital implant impressions in clinical studies. The review initial identified 6255 studies, then screened 974, which 54 were deemed as eligible but finally, 8 were included in the review. The results concluded that the accuracy of recent intra-oral scanners for digital implant impressions in patient has shown to be clinically acceptable [25]. Scanning implants in the anterior area has also shown to be more accurate than other areas, regardless of the clinician’s experience. A recent study evaluated the effect of the scanned area and operator on the accuracy of dentate arch scans with a single implant, the study scanned a dentated model with an anterior implant having a laboratory scanner as the reference scan and three operators performed the scans for complete and partial arch scans, the results indicated that trueness and precision of the scans were higher in anterior site compared with the posterior and authors concluded that the accuracy was higher in the anterior site, regardless of the scan being a partial or a complete arch, and the operator’s effect on the accuracy of partial and complete arch scans was small [26].

The implant used in this case study incorporated a platform-switching design, which has been shown to offer positive biological outcomes, such as reduced bone and tissue loss, compared to traditional platform designs. A randomized clinical trial evaluated soft tissue healing around single implants with platform switching versus traditional platform-matching implants. In the study, the implants were placed in eighteen participants, and follow-ups on the healing process were conducted at one, two, four, and six weeks, as well as at eight months. The results showed that platform switching had benefits over traditional platform matching, more specifically, lower bleeding on probing values [27]. A recent systematic review and meta-analysis also assessed platform-switching implants and their effect on bone preservation. The review analyzed randomized clinical trials and prospective studies comparing bone loss between regular and platform-switching implants. The findings indicated that platform-switching implants led to less bone loss compared to traditional platform implants [28]. Based on these advantages described in the literature, an implant with platform switching was selected for this clinical report.

The material used for the temporary restoration was polyetheretherketone (PEEK), which has shown positive results in various areas of dentistry. First, it has been recognized as a biocompatible material with tensile properties similar to those of bone, enamel, and dentin [29,30,31], making it suitable for use as a restorative material. Studies on PEEK implant abutments have shown no significant difference in bone resorption or soft tissue inflammation between PEEK and titanium implant abutments [32]. Additionally, the attachment of oral microbiota to PEEK abutments has been found to be comparable to that of titanium, zirconia, and polymethylmethacrylate abutments [33]. Due to the benefits outlined in the literature, PEEK was chosen for implant abutment fabrication.

The present case study had several limitations. A significant limitation is the low level of evidence, as it is a single case report. More clinical studies are needed to compare the clinical outcomes of immediate implants with and without soft tissue grafting procedures. In addition, further reports should clinically compare a wider range of intraoral scanner brands for final implant impressions. Future research should provide quantitative data on the success of implant therapy, such as measuring bone resorption over time, in addition to evaluating the changes in peri-implant tissue caused by the material, as well as any alterations in the ceramic restoration. Finally, longer follow-up clinical studies would provide more robust data regarding the long-term success of the treatment.

## 5. Conclusions

Immediate implant placement and restoration to replace a maxillary central incisor is an effective and predictable treatment. However, careful assessment involving a thorough review of medical and dental history, three-dimensional evaluation, digital planning, implant placement with a surgical guide, and a provisional restoration to contour the gingival tissues are essential to achieve successful outcomes.

## Figures and Tables

**Table 1:** Systematic reviews on outcomes related to immediate implant placement and loading protocols [18,19,20].

Authors and Year of Publication	Methodology	Results
Lang NP, Pun L, Lau KY, Li KY, Wong MC (2012). A systematic review on survival and success rates of implants placed immediately into fresh extraction sockets after at least 1 year [18].	Electronic search was conducted on research databases independently by the researchers. The inclusion criteria included articles in English published from 1991 to 2010. Only human studies involving endosteal dental implants with follow up times greater than 12 months were included. Implant survival, complications, and soft tissue changes were evaluated.	The annual failure rate of immediate implants was 0.82%, translating into the 2-year survival rate of 98.4%. Lower failure rates were found in groups that were provided with a course of post-operative antibiotics. Soft tissue changes occurred mostly in the first 3 months after the provision of restoration, and then stabilized towards end of the first year. Marginal bone loss predominantly took place in the first year after implant placement, with a magnitude generally less than 1 mm.
Gallucci GO, Hamilton A, Zhou W, Buser D, Chen S (2018) Implant placement and loading protocols in partially edentulous patients: A systematic review [19].	Electronic search on major research databases investigating the outcomes of different placement and loading protocols. The inclusion criteria included human studies involving endosteal implants with diameters between 3 and 6 mm with at least 10 cases and a minimum follow-up time of 12 months. A cumulative survival rate for each type of the implant placement and loading protocols was weighted by the duration of follow-up and number of implants.	Type 1A (immediate placement plus immediate restoration/loading) is a clinically documented protocol, the survival rate was 98% (median 100, range 87–100%). Type 1B (immediate placement plus early loading) is a clinically documented protocol. The survival rate was 98% (median 100, range 93–100%). Type 1C (immediate placement plus conventional loading) is a scientifically and clinically valid protocol. The survival rate was 96% (median 99, range 91–100%)
Chen R, Xu J, Wang S, Duan S, Wang Z, Zhang X, Tang Y (2024) Effectiveness of immediate implant placement into defective sockets in the esthetic zone: A systematic review and meta-analysis [20].	Electronic search was conducted independently by 2 reviewers on major research databases from from January 2000 to March 2022. A single-arm meta-analysis was performed using a statistical software program assessing the survival rates, marginal bone loss and gingival scores of dental implants placed in the esthetic zone.	The implant survival rate was 98.1%. Marginal bone loss at 6, 12, and ≥24 months were 1.03 mm and 1.15 mm. Gingival recession at 12 months was 0.25 mm. The pink esthetic score (PES) was 12.34 at 12 months and 12.58 at ≥24 months.

## Data Availability

The data presented in this study are available on request from the corresponding authors.
